# Disease progression patterns and association of prostate-specific antigen level with risk of progression in nonmetastatic castration-resistant prostate cancer

**DOI:** 10.1038/s41391-026-01076-w

**Published:** 2026-02-12

**Authors:** Alicia K. Morgans, Christopher J. D. Wallis, Susan Halabi, Andrew J. Armstrong, Patrick Adorjan, Mercedeh Ghadessi, Frank Verholen, Marc-Oliver Grimm

**Affiliations:** 1 Dana-Farber Cancer Institute, Boston, MA USA; 2 Division of Urology, Department of Surgery, University of Toronto, Toronto, ON Canada; 3 Mount Sinai Hospital, Toronto, ON Canada; 4 University Health Network, Toronto, ON Canada; 5 Duke University Division of Biostatistics, Durham, NC USA; 6 Duke Cancer Institute, Center for Prostate and Urologic Cancers, Duke University Department of Medicine, Division of Medical Oncology, Durham, NC USA; 7 Bayer Consumer Care AG, Basel, Switzerland; 8 Bayer HealthCare, Whippany, NJ USA; 9 Department of Urology, University Hospital of Jena, Jena, Germany

**Keywords:** Prostate cancer, Prostatic diseases, Cancer therapy, Outcomes research

## Abstract

**Background:**

In ARAMIS, darolutamide statistically significantly prolonged metastasis-free survival by 2 years and reduced the risk of death by 31% in patients with nonmetastatic castration-resistant prostate cancer (nmCRPC). We report post hoc analyses of ARAMIS evaluating patterns of disease progression overall and by prostate-specific antigen (PSA) response.

**Methods:**

Patients were randomized 2:1 to darolutamide (*n* = 955) or placebo (*n* = 554), with androgen-deprivation therapy (ADT). Progression included metastases on conventional imaging, PSA progression based on Prostate Cancer Working Group 2 criteria or any rise in PSA, and pain progression. Radiological progression was compared between patients who reached undetectable PSA < 0.2 ng/ml at any time and those who did not.

**Results:**

Metastatic progression occurred in 14% of patients receiving darolutamide versus 29% of patients receiving placebo, with a consistent pattern mostly isolated to bone (46%; 39%) or lymph nodes (32%; 40%). At 12 months, fewer patients receiving darolutamide versus placebo had PSA progression alone (7.8% vs. 35.9%) or both PSA and radiological progression (5.4% vs. 21.4%). Radiological progression without PSA progression occurred in 35% of metastatic events in the darolutamide group and 23% of metastatic events in the placebo group. Darolutamide led to deep PSA response (< 0.2 ng/ml) versus placebo (25.1% vs. 0.5%), and patients receiving darolutamide who reached PSA < 0.2 ng/ml experienced less radiological progression than those who did not (24 months: 8.7% vs. 33%; 36 months: 8.7% vs. 50%).

**Conclusions:**

Darolutamide plus ADT reduced the risk of metastatic progression and improved overall survival versus placebo plus ADT without changing patterns of disease progression through Month 24. Metastasis occurred without PSA progression in approximately 30% of metastatic events overall. Undetectable PSA with darolutamide was associated with reduced radiological progression that was maintained over time. These results highlight the importance of both imaging and PSA monitoring to identify disease progression in patients with nmCRPC.

## Introduction

Most patients with nonmetastatic castration-resistant prostate cancer (nmCRPC) will develop metastatic CRPC (mCRPC), which is associated with an increase in morbidity and mortality [1]. Approximately 50% of patients with nmCRPC will progress to mCRPC within 2 to 3 years, and higher prostate-specific antigen (PSA) levels and shorter PSA doubling time have been shown to predict progression to metastatic disease [2–4]. Current guidelines recommend evaluation of PSA levels every 3 to 6 months in patients with nmCRPC as a means of evaluating response to treatment and identifying metastatic progression [5–7]. Recommendations for radiological assessments (conventional imaging and/or next-generation imaging) range from routine assessments every 6 to 12 months [6] to waiting until PSA reaches 2 ng/ml to conduct a bone scan or computed tomography (CT) [7]. Symptomatic patients should undergo relevant investigations regardless of PSA level, but the need for regular assessments outside of clinical trials remains an open question. Nevertheless, patients with nmCRPC, mCRPC, or metastatic hormone-sensitive prostate cancer (mHSPC) have been reported to progress radiographically without an increase in PSA or PSA progression [8–11].

In the phase 3 ARAMIS trial, darolutamide plus androgen-deprivation therapy (ADT) statistically significantly prolonged metastasis-free survival (MFS) by nearly 2 years and reduced the risk of death by 31% versus placebo plus ADT in patients with nmCRPC [12, 13]. The incidence of adverse events was generally similar in the darolutamide and placebo groups, and few patients (< 9%) discontinued treatment due to adverse events in both groups. Using data from the ARAMIS trial, we investigated progression patterns, looking at both radiological progression and PSA progression in patients with nmCRPC. The post hoc analyses conducted had the following objectives: to evaluate prostate cancer-specific survival; the occurrence of PSA progression or pain progression before metastasis; the distribution of metastases, PSA progression, and pain progression between treatment groups; and patterns of radiological progression for all patients and for patients who did or did not achieve undetectable PSA levels (< 0.2 ng/ml).

## Methods

### Trial design and participants

ARAMIS was a randomized, double-blind, placebo-controlled phase 3 study evaluating the efficacy and safety of darolutamide in combination with ADT in patients with nmCRPC [12, 13].

Eligible patients were ≥18 years of age and had a histologically or cytologically confirmed adenocarcinoma of the prostate. Patients were required to have CRPC, a baseline PSA level of ≥2 ng/ml, a PSA doubling time of ≤10 months, and an Eastern Cooperative Oncology Group (ECOG) performance status of 0 or 1. Radionuclide bone scanning, CT, or magnetic resonance imaging were used to screen for metastases at baseline. Patients with detectable metastases or a history of metastatic disease were excluded, apart from those with pelvic lymph nodes <2 cm in diameter in the short axis below the aortic bifurcation.

Eligible patients were randomized 2:1 to receive either darolutamide 600 mg twice daily or a matched placebo with ADT during the double-blind treatment period. Randomization was stratified by PSA doubling time (≤ 6 months or >6 months) and the use of osteoclast-targeted therapy at randomization (yes or no).

### Ethics approval and consent to participate

ARAMIS was conducted in 36 countries, and the institutional review board at each center approved the study protocol. The study was conducted in compliance with the principles of the Declaration of Helsinki and in accordance with the International Conference on Harmonisation guidelines for Good Clinical Practice. All patients provided written informed consent prior to study enrollment.

### Assessments

Post-baseline metastases were identified via central review of conventional radiographic imaging every 16 weeks. Information on metastatic site was limited to the categories of lymph node, bone, or visceral metastases (at ≥1 site); data on specific sites (e.g., liver, lung) were not available. Serum PSA was measured at screening, day 1, and every 16 weeks during the double-blind treatment phase. PSA measurement was not mandatory following radiological progression. Undetectable PSA was defined as an absolute PSA level <0.2 ng/ml. PSA progression was defined according to the Consensus Guidelines of the Prostate Cancer Clinical Trials Working Group 2 (PCWG2) [14].

### Analyses

Descriptive analyses were performed using the primary data cutoff (September 3, 2018) for the double-blind period. Prostate cancer-specific survival was determined by each site based on the clinician's assessment of the cause of death. A competing risk analysis was conducted to account for other causes of death, considering standard survival analysis methods, such as Kaplan-Meier analysis, and treating patients who died of other causes as censored, thus overestimating the incidence of the outcome of interest over time. Similarly, the standard proportional hazards model may inflate the relative differences between groups, resulting in biased hazard ratios (HR).

The distribution and association between radiological progression with PSA and pain progression were evaluated. Radiological progression was defined as evidence of any radiographic disease progression (both distant and local), including new pathologic lymph nodes identified above or below the aortic bifurcation. Metastasis was determined radiographically but did not include new or progressive regional lymph nodes. PSA progression was defined in accordance with PCWG2 criteria as a ≥25% increase in PSA and an increase in absolute PSA levels ≥2 ng/ml above the nadir (i.e., lowest value recorded prior to a study visit) for patients with a decline in PSA from baseline at week 16 or above baseline for patients with no decline from baseline at week 16, confirmed by a second value obtained ≥3 weeks later [12, 14]. For this post hoc analysis, early increases in PSA levels before 16 weeks were not considered PSA progression [14]. Proportions of patients who had metastasis but did not meet PSA progression criteria based on PCWG2 criteria were analyzed for any rise in PSA prior to metastasis. Any rise in PSA was defined as an increase of >0.2 ng/ml for patients with a PSA nadir of ≤2 ng/ml and as an increase of >0.5 ng/ml for patients with a PSA nadir of >2 ng/ml prior to metastasis. Pain progression was defined as either an increase of ≥2 points from baseline in the Brief Pain Inventory Short-Form (BPI-SF) questionnaire or initiation of opioid treatment for cancer pain, whichever occurred first [12].

Baseline characteristics were summarized for subgroups based on metastasis and PSA progression. Using PSA levels and conventional imaging, the cumulative incidence of time to radiological progression was compared between patients who achieved PSA < 0.2 ng/ml and those who did not. For patients with PSA < 0.2 ng/ml who experienced radiological progression, PSA levels at the time of radiological progression were evaluated.

State sequence plots were generated by treatment group for the full analysis set, excluding patients with baseline metastases. These plots provided the proportion of patients in particular states, such as radiological progression, PSA progression, or death, at a given time.

## Results

The intent-to-treat (ITT) population of ARAMIS included 1509 patients with nmCRPC, with 955 patients in the darolutamide group and 554 patients in the placebo group [12].

### Prostate cancer-specific survival

**Fig. 1:**
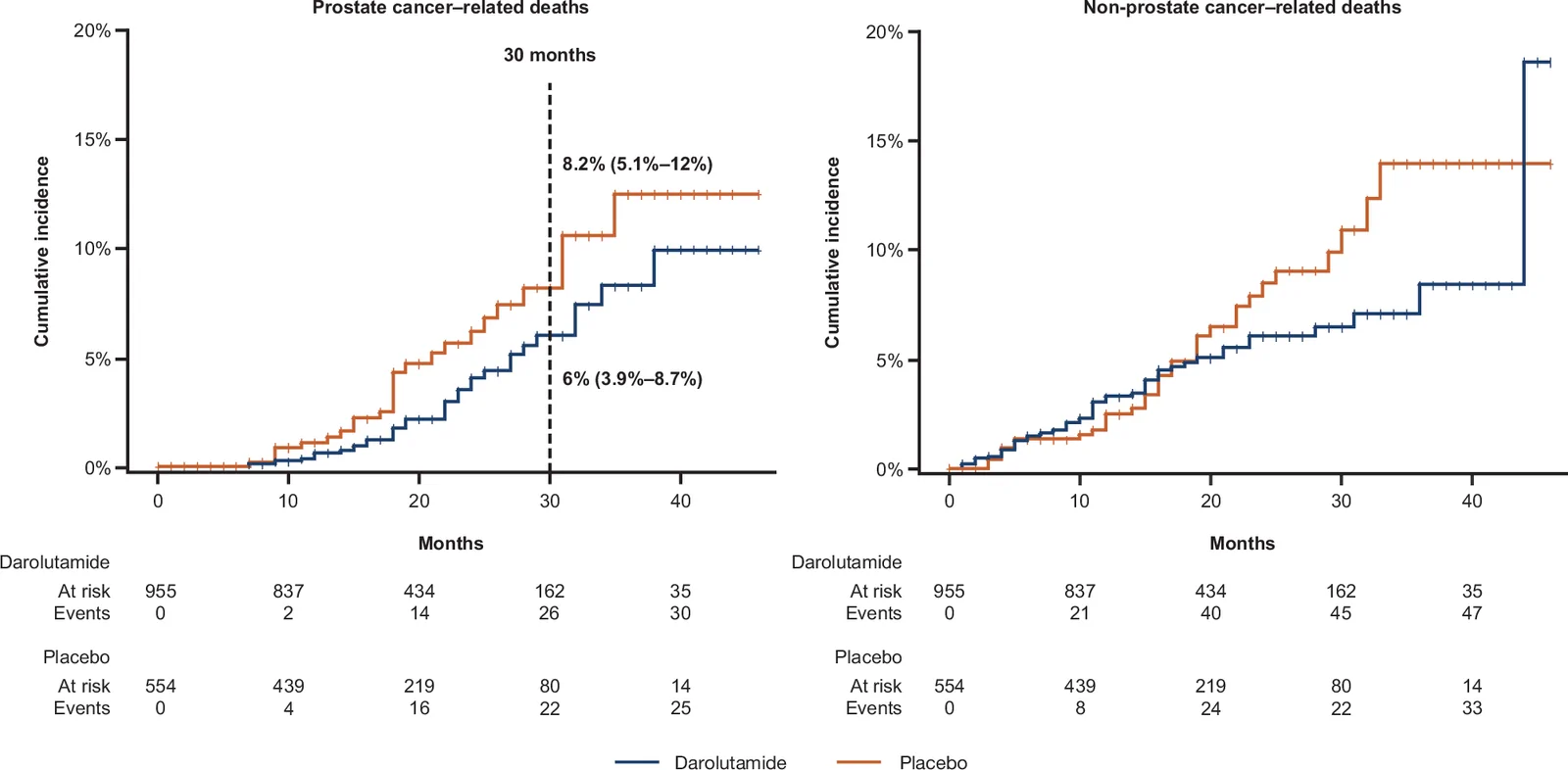
Cumulative incidence of prostate cancer-related and non-prostate cancer-related deaths. A competing risk analysis was used where the cumulative incidence is the proportion of patients who develop the outcome of interest during a specified period of time (in this case, every 10 months up to 40 months).

In ARAMIS, darolutamide significantly increased overall survival versus placebo, with a 31% reduction in the risk of death (HR 0.69, 95% confidence interval [CI] 0.53–0.88; *p* = 0.003) [13]. Notably, darolutamide reduced prostate cancer-related deaths (6.0% vs. 8.2% at 30 months), with prostate cancer the leading cause of death in both treatment arms (Fig. 1).

### Progression patterns

Darolutamide statistically significantly improved MFS versus placebo by nearly 2 years, with median (95% CI) times of 40 (34–not reached [NR]) months versus 18 (16–22) months (HR 0.41, 95% CI 0.34–0.50; *p* < 0.001) [12]. Metastases developed post-baseline in 14% (130/955) of patients receiving darolutamide versus 29% (158/554) of patients receiving placebo. At progression, the patterns of disease spread appeared similar between treatment groups. Most patients had isolated metastases in the bone (darolutamide, 46%; placebo, 39%) or distant lymph nodes (darolutamide, 32%; placebo, 40%). Visceral metastases (at ≥1 site) with darolutamide and placebo were observed in 22% and 21% of patients, respectively.

Patients who developed metastases had shorter median time from initial diagnosis to study treatment (darolutamide, 73 months; placebo, 74 months) compared with the overall ARAMIS population (86 and 84 months, respectively) (Table 1). Among patients who developed metastases, median PSA levels at baseline were similar between treatment groups (13 ng/ml and 15 ng/ml), with no discernable pattern of developing PSA progression before or after metastasis, but median (interquartile range [IQR]) PSA levels within 16 weeks of radiographic evidence of metastasis were lower in patients receiving darolutamide (17 [3.1–55] ng/ml) compared with  patients receiving placebo (48 [22–115] ng/ml). The absolute and relative median (IQR) changes in PSA levels from baseline to metastasis were −0.7 (−9.1–29) ng/ml and −0.03% (−0.8–1.8%) in the darolutamide group versus 29 (10–77) ng/ml and 1.8% (0.9–4.5%) in the placebo group. An estimate of the mean PSA levels within 16 weeks of metastasis (darolutamide, 58 ng/ml; placebo, 86 ng/ml) showed a difference (95% CI) of −28 (−54 to −1.4) ng/ml. Rates of developing bone metastases were slightly higher in patients with no PSA progression (56%) compared with those who experienced PSA progression before metastasis (34%) with darolutamide. Median time to metastasis was overall longer in patients who experienced PSA progression (darolutamide, 18.2 [range, 14.4–40.5] months; placebo, 10.9 [range, 1.1–14.8] months) before radiologic progression versus no PSA progression (darolutamide, 7.4 [range, 3.3–40.4 months]; placebo, 3.7 [range, 3.7–36.9] months).

**Table 1:** Baseline characteristics for patients with metastasis and for patients with or without PSA progression^a^ before metastasis.

	All patients with metastasis	No PSA progression^a^ before metastasis	PSA progression^a^ before metastasis			All patients		
	Darolutamide (*n* = 130)	Placebo (*n* = 158)	Darolutamide (*n* = 71)	Placebo (*n* = 57)	Darolutamide (*n* = 59)	Placebo (*n* = 101)	Darolutamide (*N* = 955)	Placebo (*N* = 554)
PSA, median (IQR), ng/ml	13 (7.5–32)	15 (6.1–27)	11 (5.2–25)	19 (8.6–32)	19 (9.5–36)	11 (6.0–23)	9.0 (4.4–20)	9.7 (4.9–21)
PSADT, median (IQR), months	3.6 (2.6–5.3)	4.2 (2.8–6.1)	3.6 (2.6–5.4)	3.8 (2.4–5.8)	3.4 (2.4–5.3)	4.8 (3.2–6.5)	4.4 (3.0–6.6)	4.7 (3.0–6.6)
ECOG PS, *n* (%)								
0	89 (69)	116 (73)	49 (69)	40 (70)	40 (68)	76 (75)	650 (68)	391 (71)
1	41 (32)	42 (27)	22 (31)	17 (30)	19 (32)	25 (25)	305 (32)	163 (29)
Gleason score, *n* (%)								
<7	22 (17)	36 (23)	15 (21)	15 (26)	7 (12)	21 (21)	217 (23)	142 (26)
≥7	104 (80)	119 (75)	54 (76)	42 (74)	50 (85)	77 (76)	711 (75)	395 (71)
Time since initial diagnosis, median (IQR), months	73 (44–108)	74 (42–116)	74 (41–101)	74 (39–106)	71 (48–112)	75 (47–120)	86 (52–127)	84 (47–134)
Prior hormonal therapy, *n* (%)								
1	24 (19)	29 (18)	14 (20)	9 (16)	10 (17)	20 (20)	177 (19)	103 (19)
≥2	99 (76)	121 (77)	52 (73)	45 (79)	47 (80)	76 (75)	727 (76)	420 (76)
N/A^b^	7 (5.4)	8 (5.1)	5 (7.0)	3 (5.3)	2 (3.4)	5 (5.0)	51 (5.3)	31 (5.6)

*ECOG PS* Eastern Cooperative Oncology Group performance status, *IQR* interquartile range, *PSA* prostate-specific antigen, *PSADT* prostate-specific antigen doubling time.

^a^PSA progression defined by Prostate Cancer Working Group 2 (PCWG2) criteria [14].

^b^Not applicable: patients who underwent surgical castration were not required to have been treated with hormonal therapy.

### Association between types of progression

For all patients who developed metastases post-baseline (darolutamide, 130/955, 14%; placebo 158/554, 29%), PSA progression by PCWG2 criteria before metastasis occurred in a total of 160 (11%) patients, occurring in fewer patients in the darolutamide group (59/130, 45%) versus those in the placebo group (101/158, 64%) (Table 2). The median (IQR) time between PSA progression by PCWG2 criteria and development of metastasis was longer for darolutamide (7.0 [3.5–11] months) versus placebo (5.6 [3.5–9.8] months). An additional 26 patients receiving darolutamide and 21 patients receiving placebo had any rise in PSA before metastasis, for a total of 85/955 (9%) and 122/554 (22%) patients having any PSA progression before metastasis, accounting for 65% and 77% of metastatic events, and 8.9% and 22% of the ITT group, in the darolutamide and placebo groups, respectively. Thus, for 35% (45/130) and 23% (36/158) of metastatic events and 4.7% (45/955) and 6.5% (36/554) of the ITT group, respectively, there was no PSA progression before metastasis.

**Table 2:** Number of patients with metastasis post-baseline, PSA progression by PCWG2 criteria, and any rise in PSA by treatment group.

	Darolutamide (*n* = 955)		Placebo (*n* = 554)	
Metastasis after baseline	130 (14% of patients)		158 (28.5% of patients)	
	**No. of patients**	**% of ITT/metastatic events**	**No. of patients**	**% of ITT/metastatic events**
PSA progression by PCWG2 criteria before metastasis	59	6.2%/45%	101	18.2%/64%
No PSA progression by PCWG2 criteria before metastasis	71	7.4%/55%	57	10.3%/36%
Any PSA progression before metastasis^a^	85	8.9%/65%	122	22%/77%
No PSA progression before metastasis	45	4.7%/35%	36	6.5%/23%

*ITT* intent-to-treat, *PCWG2* Prostate Cancer Working Group 2, *PSA* prostate-specific antigen.

^a^Includes PSA progression by PCWG2 criteria and an increase of >0.2 ng/ml for patients with a PSA nadir before metastasis of ≤2 ng/ml and as an increase of >0.5 ng/ml for patients with a PSA nadir of >2 ng/ml.

Pain progression before metastasis, which was determined by the patient-reported outcome measure BPI-SF or opioid initiation, was uncommon and similar between treatment groups (darolutamide, 17%; placebo, 18%). For both the darolutamide and placebo groups, there was no correlation observed between PSA levels, pain reported at the time of metastasis, or time to metastasis.

**Fig. 2:**
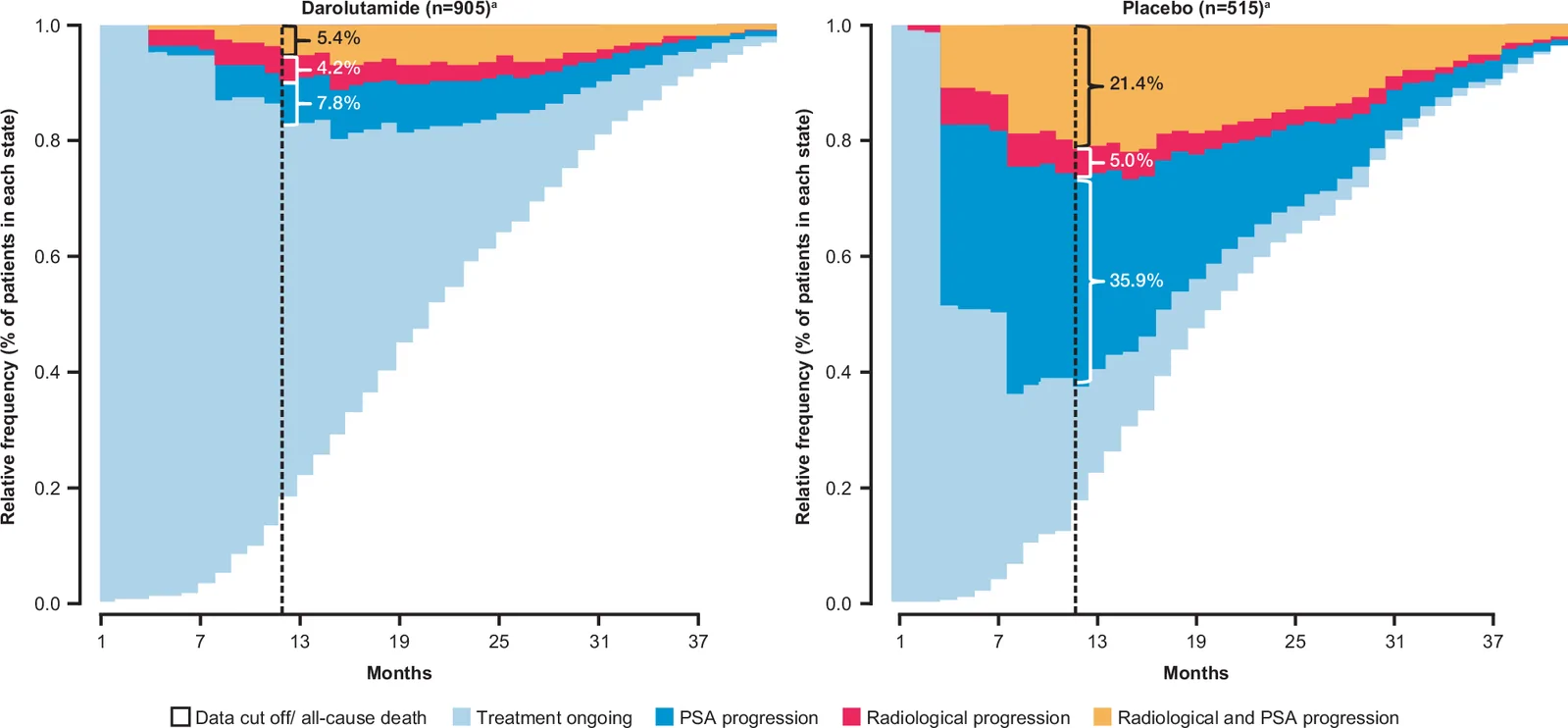
State sequence plots by treatment group showing the frequency of different disease states over time. ^a^Patients with baseline metastases were excluded. PSA prostate-specific antigen.

State sequence plots by treatment show that fewer patients receiving darolutamide versus placebo had PSA progression alone (7.8% vs. 36%) or both PSA and radiological progression (5.4% vs. 21%) at 12 months; rates of radiological progression alone were 4.2% and 5.0%, respectively (Fig. 2). The proportion of patients with progression events (PSA and/or radiological progression) was 17% in the darolutamide group versus 62% in the placebo group. Among patients receiving a placebo, progression events occurred mostly in the first 12 months. The pattern of progression remained consistent between the treatment groups over time.

### Association between PSA response and radiological progression

**Fig. 3:**
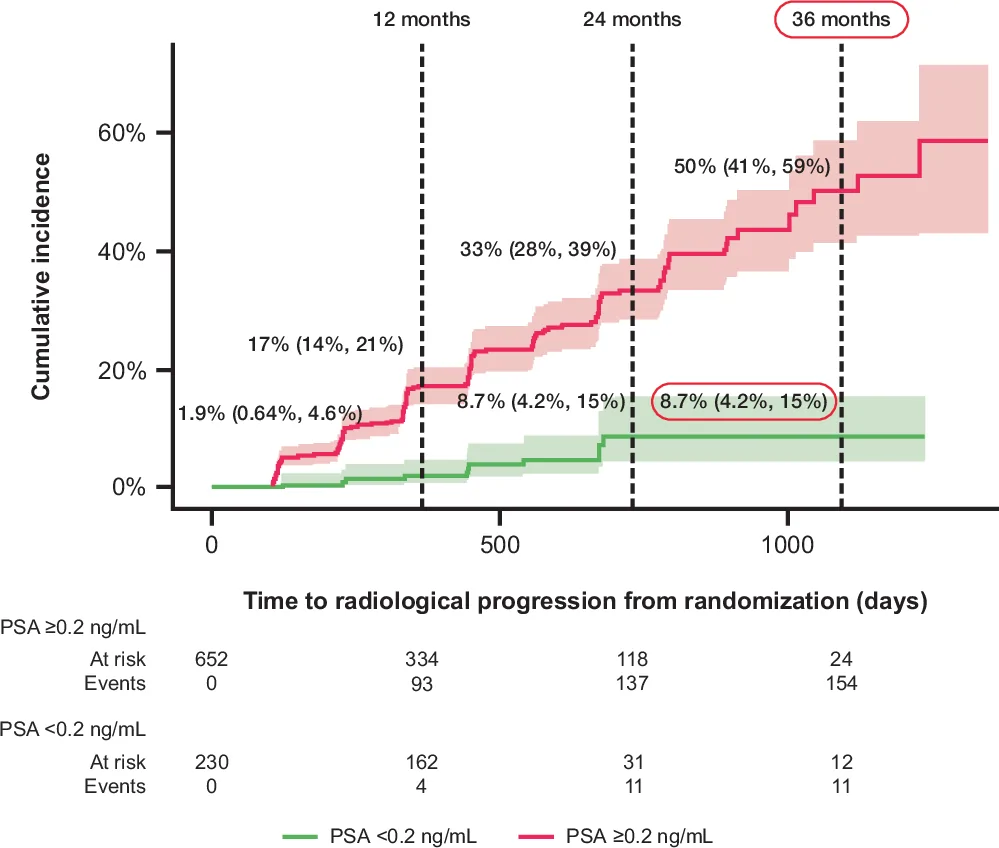
Cumulative incidence of radiological progression in patients treated with darolutamide by PSA level <0.2 ng/mL and ≥0.2 ng/mL at any time. At-risk patient numbers were calculated at the start of each time point. Randomization date is considered time zero. Shading represents 95% confidence intervals. PSA prostate-specific antigen.

Darolutamide led to deep and durable PSA responses versus placebo. As previously reported for all patients in ARAMIS, darolutamide delayed time to PSA progression (median, 33 vs. 7.3 months with placebo; HR 0.13, 95% CI 0.11–0.16) [12]. Rates of PSA < 0.2 ng/ml at any time during the study were 50-fold higher in patients receiving darolutamide versus those receiving placebo (25% vs. 0.5%). Patients receiving darolutamide who achieved PSA < 0.2 ng/ml had a lower risk of radiological progression versus those with PSA ≥ 0.2 ng/ml, with rates of 8.7% vs. 33% at 24 months (Fig. 3). At 36 months, the cumulative incidence of radiological progression remained at 8.7% for patients receiving darolutamide with PSA < 0.2 ng/ml and increased to 50% in patients with PSA ≥ 0.2 ng/ml. For the 11 patients receiving darolutamide who achieved PSA < 0.2 ng/ml and had radiological progression, PSA levels at the time of radiological progression (range, 0.02–438 ng/ml) and time to radiological progression (4–22 months) did not follow any discernible patterns, with some patients reaching PSA nadir after metastasis. Among patients who did not achieve PSA < 0.2 ng/ml at any time during the study, those receiving darolutamide versus placebo had lower PSA levels at the time of radiological progression (median [95% CI], 2.4 [2.2–2.7] vs. 3.7 [3.5–3.8] ng/ml). Baseline characteristics (Supplementary Table 1) indicate that there may have been more patients with slightly worse disease among patients who did not reach PSA < 0.2 ng/mL based on ECOG PS (ECOG PS 1; PSA ≥ 0.2 ng/mL, 33.1% vs PSA < 0.2 ng/mL, 27.4%), Gleason score ≥7 (75.2% vs 71.3%), a higher baseline median PSA (median 11.3 ng/mL vs 4.7 ng/mL), and a numerically greater number of patients with older age (75 years, 52.3% vs 40.4%) and ≥2 prior hormonal therapies (80.1% vs 67.4%).

No correlation was observed between PSA progression at the time of metastasis and overall survival (Supplementary Fig 1).

## Discussion

In patients with nmCRPC, darolutamide statistically significantly reduced the risk of death by 31% versus placebo [13]; moreover, darolutamide reduced prostate cancer-specific deaths. Findings from this post hoc analysis reaffirm that prostate cancer is the leading cause of death for patients with nmCRPC [15]. In addition, darolutamide statistically significantly reduced the risk of metastasis compared with placebo without changing the pattern of progression, as most patients in both treatment groups had isolated bone or lymph node metastasis.

Notably, metastasis was not consistently associated with PSA progression and was rarely associated with pain progression. Darolutamide was associated with deep and durable PSA responses compared with placebo. In patients receiving darolutamide who achieved PSA < 0.2 ng/ml, the risk of radiological progression was low over 24 months and did not increase through the end of the study. Furthermore, no correlation was observed with PSA progression and overall survival. However, due to the limited number of patients at risk and the very few events in each subgroup, definitive conclusions cannot be drawn from the current analysis. Among patients who developed metastasis during the study (14% receiving darolutamide and 29% receiving placebo), a smaller proportion of these patients receiving darolutamide experienced PSA progression by PCWG2 criteria or had any rise in PSA before metastasis (65%) compared with patients receiving placebo (77%). Thus, in 4.7% of patients in the darolutamide group, no PSA progression was observed, compared with 6.5% of metastatic events in the placebo group. Of all metastatic events in the darolutamide group, 55% were not associated with PSA progression by PCWG2 criteria and 35% were not associated with any PSA rise. Higher rates of developing bone metastases and a longer median time to metastasis were observed in patients who experienced no PSA progression before metastasis. Due to the lack of conclusive predictive markers, including PSA kinetics, to help identify patients who are at high risk for developing bone metastases, regular monitoring may be beneficial [16].

Our results from ARAMIS are consistent with prior reports for other androgen receptor inhibitors. In a post hoc analysis of the PROSPER study, 75 of 725 (10%) patients with nmCRPC who received enzalutamide had radiological progression without PSA progression by PCWG2 criteria, suggesting that any PSA increase in some patients did not meet the criteria threshold and imaging frequency should be investigated [9]. No increase in PSA level was observed in 29% of patients who received enzalutamide and developed metastasis. In the enzalutamide group, a higher proportion of patients with radiological progression without PSA progression had bone-only and visceral metastases compared with patients who had radiological progression with PSA progression. In the ARCHES trial of patients with mHSPC, 67% versus 43% of patients had radiographic progression without PSA progression by PCWG2 criteria, and 34% versus 15% had no rise in PSA during treatment with enzalutamide and ADT versus placebo and ADT, respectively [10]. In the TITAN trial, 52% and 17% of patients with radiological progression receiving apalutamide and ADT had no PSA progression by PCWG2 criteria or any rise in PSA; results in the placebo group were 23% and 5%, respectively [11]. Similarly, in the ENZAMET study of patients with mHSPC, radiological progression without PSA progression was observed in 114 of 1125 (10%) of patients, with similar proportions in those receiving enzalutamide (55/114 [48%]) or a standard nonsteroidal anti-androgen (59/114 [52%]) [17]. Irrespective of treatment type, 5-year overall survival rates were worse for patients with radiological progression without PSA progression (24%) compared with patients who had other types of clinical progression events, including PSA progression and treatment switch but excluding death (42%). No differences in baseline characteristics were identified between the patients with radiological progression without PSA progression versus other patients in the study. A database review of prostate cancer patients treated at the MD Anderson Cancer Center from 1999 to 2004 identified 46 patients with metastasis and concomitant PSA < 2 ng/ml [18]. Atypical histological variants of prostate cancer were found in 21 of these patients, and most had a Gleason score >7 at diagnosis.

Thus, the results of our analysis showing that radiological progression can occur in the absence of PSA progression, particularly in the setting of an androgen receptor pathway inhibitor plus ADT therapy, provide further support for the use of regular imaging to monitor patients with nmCRPC. Despite recommendations for routine monitoring of patients with nmCRPC [6, 7], a retrospective, longitudinal, population-based study of 944 patients in Canada found underutilization of both PSA testing and imaging, even among patients with high-risk disease defined as PSA doubling time of <10 months[19]. When taking into consideration the median time to radiographic progression with PSA reported here in ARAMIS, imaging every 3–6 months during the first year of treatment and 6–12 months thereafter is reasonable to implement for patients with nmCRPC. This may increase the chance of detecting events associated with poor prognosis that may occur in the absence of symptoms (ie, no pain progression despite the development of metastasis) [8, 9, 20, 21].

One possible explanation for why some patients may progress radiographically without PSA progression is the emergence of lineage plasticity and neuroendocrine prostate cancer (NEPC) after androgen receptor-targeted therapy [22, 23]. Molecular alterations and clinical characteristics of NEPC include loss of the androgen receptor and androgen-regulated protein expression, genomic instability, visceral metastases, and low PSA values. Alternatively, androgen receptor inhibitors might have a strong effect on PSA expression despite early disease progression. The ARAMIS study design is limited in that it did not include evaluation of genetic data or post-progression biopsies, and assessment of PSA levels was not mandatory after radiological progression occurred. Thus, we cannot exclude lineage plasticity or the development of NEPC in these patients who progressed to mCRPC [24]. Our analysis is prone to bias by analyzing a post-baseline characteristic, PSA response, which may have been influenced by study treatment. Patients who responded to treatment and reached undetectable PSA had a longer time until PSA progression and metastasis. PSA progression can also be affected by other factors, such as previous radiotherapy, prior prostatectomy, and ethnicity, which were not controlled for in this analysis [25, 26]. Studies are needed to assist in the identification of patients who have radiological progression without PSA progression or with low PSA levels, and the findings of this analysis should be considered in updates to PCWG progression criteria.

In conclusion, darolutamide was associated with lower rates of PSA and/or radiological progression in patients with nmCRPC compared with placebo. Patients who achieved undetectable PSA < 0.2 ng/ml had a low risk of radiological progression throughout the study; however, radiological progression occurred in the absence of PSA progression. These results emphasize the need for both imaging and PSA monitoring to identify disease progression in patients with nmCRPC.

## Supplementary information

Supplemental Materials

## Data Availability

Availability of the data underlying this publication will be determined according to Bayer’s commitment to the EFPIA/PhRMA “Principles for responsible clinical trial data sharing”. This pertains to the scope, time point and process of data access. As such, Bayer commits to sharing upon request from qualified scientific and medical researchers patient-level clinical trial data, study-level clinical trial data, and protocols from clinical trials in patients for medicines and indications approved in the United States (US) and European Union (EU) as necessary for conducting legitimate research. This applies to data on new medicines and indications that have been approved by the EU and US regulatory agencies on or after January 01, 2014. Interested researchers can use www.vivli.org to request access to anonymized patient-level data and supporting documents from clinical studies to conduct further research that can help advance medical science or improve patient care. Information on the Bayer criteria for listing studies and other relevant information is provided in the member section of the portal. Data access will be granted to anonymized patient-level data, protocols and clinical study reports after approval by an independent scientific review panel. Bayer is not involved in the decisions made by the independent review panel. Bayer will take all necessary measures to ensure that patient privacy is safeguarded.
