# Supplementary material for: Disease progression patterns and association of prostate-specific antigen level with risk of progression in nonmetastatic castration-resistant prostate cancer
Source: Prostate Cancer Prostatic Dis. 2026 Feb 12;29(4):806–12. doi: 10.1038/s41391-026-01076-w (PMC13623639; doi:10.1038/s41391-026-01076-w)
Supplement: Supplementary file 1 [file 41391_2026_1076_MOESM1_ESM.docx]

**Supplementary Materials**

Contents

Supplementary Fig 1 Cumulative incidence of deaths in patients treated with darolutamide or placebo by PSA progression………………………………………………………………………………………………………………………………….2

Supplementary Table 1. Demographics and baseline characteristics for patients treated with darolutamide by PSA level <0.2 ng/mL and ≥0.2 ng/mL at any time…………………………………………………………………………………………………………………………………………………………3

**Supplementary Fig 1** **Cumulative incidence of deaths in patients treated with darolutamide or placebo by PSA progression.** At-risk patient numbers were calculated at the start of each timepoint.

NR, not reached; PSA, prostate-specific antigen.

**Supplementary Table 1.** Demographics and baseline characteristics for patients treated with darolutamide by PSA level <0.2 ng/mL and ≥0.2 ng/mL at any time.

|  | Darolutamide | |
| --- | --- | --- |
|  | **PSA ≥0.2 ng/mL (n=652)** | **PSA <0.2 ng/mL (n=230)** |
| Age, median (range) | 75 (69–80) | 73 (68–78) |
| Age category, n (%) |  |  |
| <65 years | 79 (12.1) | 22 (9.6) |
| 65–74 years | 232 (35.6) | 115 (50.0) |
| ≥75 years | 341 (52.3) | 93 (40.4) |
| PSA, median (IQR), ng/ml | 11.3 (5.5–21.9) | 4.7 (2.9–8.8) |
| PSADT, median (IQR), months | 4.4 (3.1–6.7) | 4.5 (3.0–6.6) |
| ECOG PS, n (%) |  |  |
| 0 | 436 (66.9) | 167 (72.6) |
| 1 | 216 (33.1) | 63 (27.4) |
| Gleason score, n (%) |  |  |
| <7 | 138 (21.2) | 64 (27.8) |
| ≥7 | 490 (75.2) | 164 (71.3) |
| Time since initial diagnosis, median (IQR), months | 92.5 (55.3–129.4) | 79.4 (45.0–120.2) |
| Prior hormonal therapy, n (%) |  |  |
| 1 | 108 (16.6) | 53 (23.0) |
| ≥2 | 522 (80.1) | 155 (67.4) |
| N/A^a^ | 22 (3.4) | 22 (9.6) |

Full analysis set. ^a^ Not applicable: patient who underwent surgical castration were not required to have been treated with hormonal drug therapy.

ECOG PS, Eastern Cooperative Oncology Group performance status; IQR interquartile range; PSA, prostate-specific antigen; PSADT, prostate-specific antigen doubling time.
